# Determining the Epidemiologic Aspects and Treatment Results of Patients with Idiopathic Granulomatous Mastitis (IGM) Visiting Rheumatology and Surgery Clinics Using Fuzzy Artificial Intelligence, from 2015 to 2023: Treatment Results of Patients with Idiopathic Granulomatous Mastitis Using Fuzzy Artificial Intelligence

**DOI:** 10.31661/gmj.vi.3801

**Published:** 2025-07-01

**Authors:** Maryam Masoumi, Pouya Derakhshan-Barjoei, Fatemeh Mollarahimi-Maleki, Mojdeh Bahadorzadeh, Mohammad Shafaei

**Affiliations:** ^1^ Department of Clinical Research and Development Unit, Qom University of Medical Sciences, Qom, Iran; ^2^ Artificial Intelligence and Data Analysis Research Center, Department of Electrical Engineering, SR.C., Islamic Azad University, Tehran, Iran; ^3^ Department of Community and Family Medicine, School of Medicine, Spiritual Health Research Center, Qom, Iran; ^4^ Department of Surgery, Qom University of Medical Sciences, Qom, Iran; ^5^ Qom University of Medical Sciences, Qom, Iran

**Keywords:** Granulomatous Mastitis, Treatment, Epidemiology, Fuzzy Artificial Intelligence

## Abstract

**Background:**

Granulomatous mastitis (GM) is a benign inflammatory disease that affects the breasts. From a pathological perspective, GM is characterized by chronic granulomatous and necrosing lesions containing small abscesses and inflammation of lobules. A variety of cures has been listed for this condition, including follow-up without intervention, antibiotic therapy, and consumption of corticosteroids, drainage, excisions, and mastectomy, but still the best cure remains unknown. This study aims to determine the epidemiologic aspects and treatment results of patients with idiopathic GM visiting rheumatology and surgery clinics from 2015 to 2023.

**Materials and Methods:**

This retrospective cohort study analyzed 39 patients with IGM visiting rheumatology and surgery clinics from 2015 to 2023. Based on a study by Kehribar et al., granulomatous mastitis has an annual prevalence of 2.4 in 100,000 cases and an incidence rate of 0.37% [1]. The required sample size was calculated as 39 people. Data were collected using a census method, categorized into types of treatment and response to treatment and patient characteristics, with ethical approval obtained.

**Results:**

The study analyzed 39 patients with an average age of 34.48±5.47 years, ranging from 22 to 45 years. Treatment strategies varied: oral steroids (25 patients), antibiotics (9 patients), surgical treatment (9 patients), combined antibiotic and surgical treatment (4 patients), steroids and MTX (2 patients), and combined steroid and antibiotic treatment (15 patients). Disease recurrence was noted in 15.4% of patients. Recovery outcomes were no recovery in 7 patients, partial recovery in 15 patients, and complete recovery in 17 patients.

**Conclusion:**

The results found that the type of treatment has no statistically significant relationship with the patient’s recovery process. Complete recovery was higher in the oral steroid and steroid plus antibiotic treatment group compared to other methods. Using AI to investigate and evaluate treatments for granulomatous mastitis can provide valuable insights into the effectiveness and safety of various therapeutic approaches. By leveraging machine learning and AI techniques, researchers and clinicians can make more informed decisions that ultimately improve patient outcomes.

## Introduction

Idiopathic granulomatous mastitis is a rare inflammatory disease of the breast, for which there is a lack of consensus on the treatment protocol [1]. Granulomatous mastitis (GM) is a benign inflammatory disease that affects the breasts. It was first introduced by Kessler and Wolloch in 1972 [2] . GM is frequently seen in young women with a history of breastfeeding and its most common manifestation is a hard mass, one-sided, and with obvious borders in breasts which is associated with the inflammation of the skin of the breast.

From a pathological perspective, GM is characterized by chronic granulomatous and necrosing lesions containing small abscesses and inflammation of lobules.

To diagnose IGM, other well-known conditions such as Tuberculosis, Sarcoidosis, and parasitic infections that display a similar histologic view must be ruled out. The probable cause of GM appears to be an autoimmune reaction to secreted material from mammary ducts. Additionally, pregnancy, breastfeeding, oral contraceptive pill consumption, and certain infections can also cause this condition.

. The mammography view in most cases resembles a breast carcinoma but a sonography representing a considerable amount of hypoechoic and connected lesions is a better indication of GM. The significant matter in this condition is that due to its resemblance between the radiologic and clinical aspects, it is considered a suspicious or malignant lesion of the breast in more than half of the early diagnosis cases.

A variety of cures has been listed for this condition including follow-up without intervention, antibiotic therapy, consumption of corticosteroids, drainage, excision, and mastectomy but still the best cure remains unknown.

Even though GM is considered benign, its early and accurate diagnosis can be significant from many aspects: 1. Its treatment and diagnosis procedure is still a mystery on which there is no agreement. 2. Due to its imitation of cancerous symptoms, especially inflammatory cancers, a misjudged diagnosis can result in financial and psychological expenses for both the system and the patient and may even lead patients to an unnecessary mastectomy. 3. On the other hand, because of the lack of an approved treatment protocol we would face many complications of experimental treatment such as allergic reactions to antibiotics followed by unnecessary surgical interventions. The goal of this study is to determine the epidemiologic aspects and treatment results of patients with idiopathic GM visiting rheumatology and surgery clinics from 2015 to 2023.

## Materials and Methods

**Table:** Table1. Response to Treatment, Partial Response (Improvement in All Clinically Significant Symptoms, Including Pain, Swelling, Erythema, and Induration) or Complete Response (Complete Resolution of the Aforementioned Symptoms)

P-VALUE	Complete Response	Partial Response	No Response	
0,979	(15,4) 6	(5,1) 2	(2,6) 1	Antibiotic
0,077	(33,3) 13	(17,9) 7	(12,8) 5	Oral steroid
0,398	(17,9) 7	(5,1) 2	(0) 0	Surgery
0,554	(5,1) 2	(0) 0	(0) 0	Steroid and MTX
0,514	(28,2) 11	(5,1) 2	(5,1) 2	Steroid plus antibiotic
0,287	(10,3) 4	(0) 0	(0) 0	Surgery plus antibiotic

**Figure-1:**
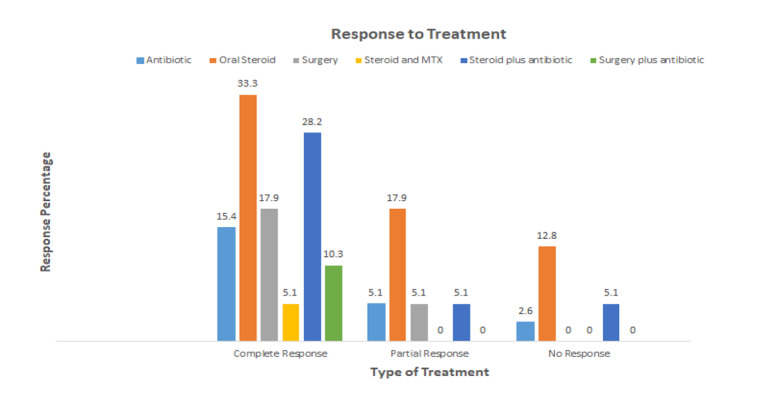
Figure 1. Various treatment strategies and response to treatment

This study was conducted on all patients with Granulomatous mastitis (GM) visiting rheumatology and surgery clinics from 2015 to 2023.39 patients were ultimately selected through a census method. The ethical approval obtained from the Ethics Committee of Qom University of Medical Sciences (IR.MUQ.REC1402.217) Data collected and documented in the prepared checklists while maintaining confidentiality and adhering to ethical guidelines. In all these patients, after microbiology, pathology, and rheumatology investigations, the underlying causes were rejected and the idiopathic nature of the disease was proved. The most important differential diagnosis in idiopathic granulomatous mastitis was tuberculosis and sarcoidosis infections. To rule out these cases, chest x-ray lung CT scan PCR for tuberculosis, and culture of secretions were necessary to rule out infection. Next, the demographic information, clinical and radiological manifestations, pathology report, and the type of treatment performed were recorded from the patient's files. Also, the recorded follow-ups of all patients in the file were checked for the rate of response to treatment and recurrence in treatment groups. The response to treatment in the form of clinical and radiological improvement was followed up by conducting control ultrasounds to obtain more detailed information by making a phone call about the latest situation.

The patient was informed. Finally, all the data were entered into SPSS software version 22 (IBM SPSS Statistics version 22-USA) and analyzed with appropriate statistical tests also evaluate the intelligent method using MATLAB and Phyton softwares (Microsoft MATLAB R2022b and Phyton 3.12.3) for self-learning and rule extraction for the fuzzy algorithm [3][4][5][6][7][8].

In this study, to compare the treatment groups, independent t-test, and chi-square test were used by P-value (significant less than 0.05) to test a hypothesis about a parameter. Our proposed AI methodology aims to systematically analyze the epidemiological aspects and treatment results of patients with idiopathic granulomatous mastitis through a combination of data collection, preprocessing, machine learning, and statistical analysis. Using fuzzy AI instead of traditional AI is beneficial because of the inherent uncertainty and ambiguity present in the data. Considering subjectivity in assessment, disease severity and treatment response often involves subjective clinical judgments.

For example, assessing the extent of inflammation or a patient's functional capacity can be imprecise and vary between clinicians. Fuzzy logic can model this subjectivity by assigning degrees of membership to different severity levels or response categories. This study employed a fuzzy inference system (FIS) to analyze epidemiological and treatment outcome data from patients with GM. In this algorithm data preprocessing is consisted of types of treatment and response of it. The dataset was split into training (70%), validation (15%), and testing (15%) sets. The model achieved an accuracy of 75%, precision of 81%, specificity of 70%, sensitivity of 87% and AUC ( Area Under the Curve) of 0.74.Also, Triangle membership functions were considered for the response rate and epidemiology in a Mamdani interference system. Due to the rare incidence of this disease, our artificial intelligence algorithm was faced with limited data so according to explorative results, this model can be used for future studies applying big data.

## Results

**Figure-2:**
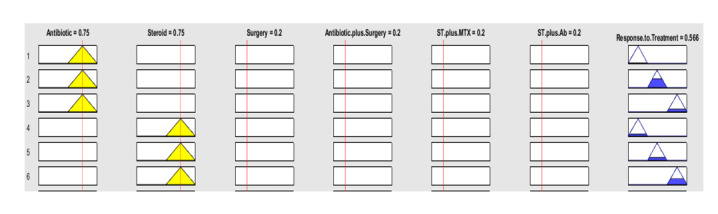
Figure 2. Typical Rule of Response of Treatment for Sample Case

**Figure-3:**
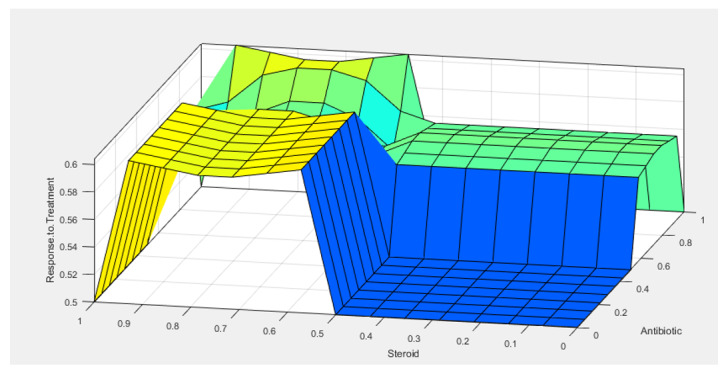
Figure 3. Response to Treatment Behavior for Sample Case

The study analyzed 39 patients with an average age of 34.48 ± 5.47 years, ranging from 22 to 45 years. Most patients (94.9%) were married, while 5.1% were single. The average age of their last child was 3 ± 1.7 years. The mean time from diagnosis to treatment was 4.36 ± 2.53 years. A significant majority (87.2%) had a history of breastfeeding, and 48.7% were using oral contraceptive pills (OCP). Family history of cancer was observed in 5.1% of patients, with none reporting previous breast trauma. The average number of children per patient was 2.35 ± 1.03, and the average mass size was 21 ± 16.63 mm. The involvement frequency was 3.3% on the right side, 38.5% on the left side, and 28.2% bilaterally. General symptoms were present in 94.2% of patients, with systemic symptoms in 12 patients. Symptom frequencies included pain (64.1%), swelling and redness (23.1%), mass palpation (92.3%), and pain with nipple discharge (59%). Diagnostic methods utilized were ultrasound in 82.1% and mammography in 17.9% of cases. Treatment strategies varied: oral steroids (25 patients), antibiotics (9 patients), surgical treatment (9 patients), combined antibiotic and surgical treatment (4 patients), steroids and MTX (2 patients), and combined steroid and antibiotic treatment (15 patients). Disease recurrence was noted in 15.4% of patients. Recovery outcomes were no recovery in 7 patients, partial recovery in 15 patients, and complete recovery in 17 patients. Based on the results obtained from Table-1, it was found that the type of treatment has no statistically significant relationship with the recovery process of the patients, so as the results show, complete recovery was higher in the oral steroid treatment group, steroid plus antibiotic compared to other methods. In Figure-1, response to different treatment is illustrated. Also, the relation between changing in parameters and response to treatment from fuzzy interference system is shown in Figures-2 and -3.

## Discussion

Idiopathic granulomatous mastitis is a rare, chronic, benign inflammatory breast disease. Its cause was unknown, and it was hypothesized that local autoimmune response to residual or extruded fat or protein in the breast ducts during reproductive age due to previous hyperprolactinemia may be responsible[9]. The response to steroids supports this autoimmune nature of the disease [10]. As in our study, most patients responded to steroid therapy. Idiopathic granulomatous mastitis is a non-malignant inflammatory process of the breast that was first described in 1972 by Kessler and Wolloch [2]. Idiopathic granulomatous mastitis (IGM) is an uncommon benign disorder that can mimic breast carcinoma and breast abscess[11]. Although this disease is rare worldwide, according to the study by Altinotopark et al., it appears to have a higher prevalence in Iran and neighboring countries such as Turkey and Saudi Arabia [12][13]. In the present study, only 39 patients with diagnosed granulomatous mastitis were evaluated over several years, indicating a low prevalence of this disease.

This disease is clinically and radiologically mimicking breast cancer, to the extent that relying solely on radiological findings in ultrasound, mammography, and MRI cannot definitively differentiate it from breast cancer [14]. This mistake could potentially cause a significant psychological. The burden on the individual and their family, and in the case of incorrect mastectomy or partial mastectomy, may lead to irreversible consequences.

However, histological features of immune-mediated inflammation, such as vasculitis and primarily plasma cells and lymphoid aggregates, are not observed in IGM Idiopathic granulomatous mastitis is not typically associated with trauma, specific infections, or foreign materials [14].

Idiopathic granulomatous mastitis is mostly seen in postmenopausal women and often in women of reproductive age. The average age at presentation in our study was 34 years. However, this age range is comparable to the results reported in other studies, which reported an age range between 33.5 to 39 years [15][16]. The most common manifestation was a unilateral breast mass with or without pain. Bilateral cases were less frequent, reported in 28.2% of cases. Since all patients in this study had a history of childbirth and almost all had a history of breastfeeding, it can be said that breastfeeding is a major predisposing factor for this condition, which has also been confirmed in previous studies. The use of contraceptive pills is mentioned; in various studies, the rate of contraceptive pill use among patients has ranged from 0 to 42% [13].

Steroid therapy should be considered based on the idea that this is an autoimmune disease like IGM [17]. For example, in another study, the rate of contraceptive pill use in patients with idiopathic granulomatous mastitis was 28% [18]. In our study, the history of contraceptive drug use was 48.7%, but most patients did not mention contraceptive pill use at the onset of symptoms, which, alongside previous study results, diminishes the role of this factor in the pathogenesis of idiopathic granulomatous mastitis.

Various therapeutic strategies have been suggested for idiopathic granulomatous mastitis in different studies. These strategies include patient monitoring for self-improvement, corticosteroids, methotrexate, surgical options such as lumpectomy, incomplete mastectomy, complete mastectomy, and drainage [12][19].

None of these treatment modalities are recommended as the gold standard yet. In various studies, there was no significant difference in recurrence rates between different methods. Atak et al., as well as Kayahan et al., believe that due to faster improvement, fewer side effects, and the possibility of definitive diagnosis, surgical treatment for removing the lesion is preferable [20][21].

In contrast, in some studies, such as the study by Pandey et al., oral steroid therapy is considered a suitable non-surgical conservative treatment for breast-preserving management in patients with idiopathic granulomatous mastitis [12]. In the study of Sheybani et al., treatment with prednisolone and methotrexate with or without surgery has been suggested as the treatment of choice in patients with idiopathic granulomatous mastitis [22]. In the study of Asieh sadat Fattahi et al. The overall recurrence rate was 17.18% in IGM treatments [23]. Various treatments were used in our study for current patients, including oral steroid therapy, surgery (including drainage and mass removal) along with oral steroids, oral steroids, and methotrexate, oral steroids along with antibiotics, antibiotic therapy alone, surgery along with antibiotic therapy, surgery (including drainage and mass removal), surgery along with oral steroids and antibiotics. Overall, a 15.4% recurrence rate was observed across different treatment modalities. Out of 39 included in the study, 7 patients did not show improvement, 15 patients had partial improvement, and 17 patients had complete improvement. Additionally, it was found that the type of treatment did not have a statistically significant correlation with the patient's improvement, although the results indicate that complete improvement was higher in the oral steroid and surgical treatment group compared to other methods. The integration of artificial intelligence (AI) into medical research and treatment methodologies has gained significant traction, particularly in the context of managing idiopathic granulomatosis (GM). The study conducted at Qom University of Medical Sciences exemplifies how AI can enhance decision-making processes based on clinical outcomes. AI's ability to analyze historical data allows for better predictive modeling regarding treatment outcomes. By recognizing complex associations within the data, AI can assist clinicians in making informed decisions about potential treatments based on the likelihood of success for similar cases. The empirical results have shown that the proposed algorithm is an available and effective approach for our fuzzy rule extraction problem.

## Conclusion

The results found that the type of treatment has no statistically significant relationship with the patient's recovery process. Complete recovery was higher in the oral steroid and steroid plus antibiotic treatment group compared to other methods. Using AI to investigate and evaluate treatments for granulomatous mastitis can provide valuable insights into the effectiveness and safety of various therapeutic approaches. Artificial intelligence method has been applied to obtain the relationship between parameters and response to treatment. Rules of the fuzzy logic system have been managed by AI output. Our proposed joint method obtained an assessment and prediction of the response to treatment process. However, because of low incidence prevalence of the disease and the small sample size, this method has been validated for large-scale data in future studies.

## Conflict of Interests

The authors declare that they have no competing interests.

## References

[R1] Kehribar D, Duran T, Polat A, Ozgen M (2020). Effectiveness of methotrexate in idiopathic granulomatous
mastitis treatment. The American Journal of the Medical Sciences.

[R2] Kessler E, Wolloch Y (1972). Granulomatous mastitis: a lesion clinically simulating
carcinoma. American journal of clinical pathology.

[R3] Foroozandeh E, Derakhshan-Barjoei P, Bahadorzadeh M (2018). Investigation the effect of emotional control and extroversion on
severity of central serous retinopathy in patients using fuzzy logic
algorithm. Journal of Health and Biomedical Informatics.

[R4] Foroozandeh E, Derakhshan-Barjoei P, Bahadorzadeh M (2018). Fuzzy Logic Evaluation of Personality Profile and Alexithymia
with Emotional Suppression in Patients with Central Serous Retinopathy. International Journal of Research.

[R5] Abad MJ, Derakhshan-Barjoei P (2012). Heuristic model of cellular learning automata for fuzzy rule
extraction. Research Journal of Applied Sciences, Engineering and Technology.

[R6] Bahadorzadeh M, Vahedian M, Khan Babaei, Derakhshan-Barjoei P (2023). The study of predisposing factors related to perforation in
patients with peptic ulcer in shahid beheshti hospital using fuzzy logic,
qom, during 2019 to 2022. Tehran University of Medical Sciences Journal.

[R7] Hosseini SJ, Derakhshan-Barjoei P, Bahadorzadeh M, Seifaddini A, Vahedian M (2024). Efficacy of Oral Gabapentin and Acetaminophen for Postoperative
Analgesia in Anorectal Surgery: A Fuzzy Logic Evaluation. Middle East Journal of Digestive Diseases.

[R8] Derakhshan-Barjoei P, Bahadorzadeh M (2012). Enhancement in medical image processing for breast calcifications
and tumor detection. Research Journal of Applied Sciences, Engineering and Technology.

[R9] Dilaveri C, Degnim A, Lee C, DeSimone D, Moldoveanu D, Ghosh K Idiopathic granulomatous mastitis. The Breast Journal.

[R10] DeHertogh DA, Rossof AH, Harris AA, Economou SG (1980). Prednisone management of granulomatous mastitis. New England Journal of Medicine.

[R11] Patel RA, Strickland P, Sankara IR, Pinkston G, Many W, Rodriguez M (2010). Idiopathic granulomatous mastitis: case reports and review of
literature. Journal of general internal medicine.

[R12] Pandey TS, Mackinnon JC, Bressler L, Millar A, Marcus EE, Ganschow PS (2014). Idiopathic granulomatous mastitis—a prospective study of 49 women
and treatment outcomes with steroid therapy. The breast journal.

[R13] Altintoprak F, Kivilcim T, Ozkan OV (2014). A etiology of idiopathic granulomatous mastitis. World Journal of Clinical Cases: WJCC.

[R14] Fletcher A, Magrath IM, Riddell RH, Talbot IC (1982). Granulomatous mastitis: a report of seven cases. Journal of clinical pathology.

[R15] Patel RA, Strickland P, Sankara IR, Pinkston G, Many W, Rodriguez M (2010). Idiopathic granulomatous mastitis: case reports and review of
literature. Journal of general internal medicine.

[R16] Yildiz S, Aralasmak A, Kadioglu H, Toprak H, Yetis H, Gucin Z, Kocakoc E (2015). Radiologic findings of idiopathic granulomatous mastitis. Medical Ultrasonography.

[R17] Özel L, Ünal A, Ünal E, Kara M, Erdoğdu E, Krand O, Güneş P, Karagül H, Demiral S, Izzet Titiz (2012). Granulomatous mastitis: is it an autoimmune disease Diagnostic
and therapeutic dilemmas. Surgery today.

[R18] Al-Khaffaf B, Knox F, Bundred NJ (2008). Idiopathic granulomatous mastitis: a 25-year experience. Journal of the American College of Surgeons.

[R19] Cheng J, Du YT, Ding HY (2010). Granulomatous lobular mastitis: a clinicopathologic study of 68
cases. Zhonghua Bing li xue za zhi= Chinese Journal of Pathology.

[R20] Kayahan M, Kadioglu H, Muslumanoglu M (2012). Management of patients with granulomatous mastitis: analysis of
31 cases. Breast Care.

[R21] Boufettal H, Essodegui F, Noun M, Hermas S, Samouh N (2012). Idiopathic granulomatous mastitis: a report of twenty cases. Diagnostic and interventional imaging.

[R22] Sheybani F, Sarvghad M, Naderi H, Gharib M (2015). Treatment for and clinical characteristics of granulomatous
mastitis. Obstetrics & Gynecology.

[R23] Fattahi AS, Amini G, Sajedi F, Mehrad-Majd H (2023). Factors Affecting Recurrence of Idiopathic Granulomatous
Mastitis: A Systematic Review. The Breast Journal.

